# Floating photocatalyst of B–N–TiO_2_/expanded perlite: a sol–gel synthesis with optimized mesoporous and high photocatalytic activity

**DOI:** 10.1038/srep29902

**Published:** 2016-07-19

**Authors:** Hongbo Xue, Ya Jiang, Kechun Yuan, Tingting Yang, Jianhua Hou, Chuanbao Cao, Ke Feng, Xiaozhi Wang

**Affiliations:** 1 School of Environmental Science and Engineering, Yangzhou University, Yangzhou 225127, P.R. China; 2 Center of Materials Science, Beijing Institute of Technology, Beijing 100081, P.R. China

## Abstract

Optimized mesoporous photocatalyst endowed with high specific surface area and large pore size was synthesized by sol–gel method. These large pore mesoporous materials (33.39 nm) were conducive to the movement of larger molecules or groups in pore path and for effective use of active sites. The high specific surface area (S_BET_, 99.23 m^2^ g^−1^) was beneficial to catalytic oxidation on the surface. Moreover, B and N co-doped anatase TiO_2_ in the presence of Ti–O–B–N and O–Ti–B–N contributed to the pore structure optimization and enhanced photoresponse capacity with a narrow band gap and red shift of absorption. The obtained materials with floating characteristics based on expanded perlite (EP) showed favorable features for photocatalytic activity. The best RhB photodegration rate of B–N–TiO_2_/EP (6 mg/g, 24 wt% TiO_2_) reached 99.1% after 5 h in the visible region and 99.8% after 1 h in the UV region. The findings can provide insights to obtain floatable photocatalysts with simple preparation method, optimized mesoporous, co-doping agents, as well as good photocatalytic performance, coverable and reusability. B–N–TiO_2_/EP has potential applications for practical environmental purification.

Migration of organic compounds from chemical industries, manufacture and agriculture into water, air and soil has attracted global attention. Photocatalytic oxidation with titanium dioxide (TiO_2_) has been considered to degrade organic containments effectively^1^,^2^. Mesoporous TiO_2_ materials generally have remarkable properties, such as large and uniform pore sizes, porous structures and a higher electron–hole separation rate; these materials are also known for its worldwide availability, chemical stability, high photocorrosion stability and environmental friendliness^3^,^4^. Furthermore, mesoporous TiO_2_ possess potential in photoelectrochemical water splitting and photochemical catalysis, especially the high crystallinity of anatase play the important role^5^,^6^. Investigations on extending optical response capacity to visible light and gaining high surface area are urgently needed due to the band gap of anatase TiO_2_ limits the utilization of visible light, and the low absorption and reaction rate are caused by the low surface area of normal TiO_2_^7^.

In recent years, many approaches have been conducted to dope impurities, such as TiO_2_ doped with nonmetallic elements (*e.g.*, B, C, N, F, and S). Among the co-doping atoms, B–N co-doping is considered the most effective. Xu *et al*.^8^ reported that B–N–TiO_2_ exhibits the higher photocatalytic activity than porous N–doped TiO_2_ and unmodified TiO_2_ film under UV and visible lights. Zhang *et al*.^7^ found that the absorption band edge of B–N co-doped sample exhibits an evident red shift and a synergistic effect of two dopants that enhance photocatalytic activity. Gopal *et al*.^9^ reported that B fills oxygen vacancy and N serves as a paramagnetic probe.

However, these powdered composite materials can also lead to several problems as follows: (i) The derived powder aggregates easily in suspension, thereby resulting in low utilization rate. (ii) The filtration or separation process of the particles is challenging. (iii) The powder is difficult to apply to a continuous flow system. (iv) Although the sizes of mesoporous sheet- and flower-like TiO_2_ films could be enlarged to 20–30 nm, the accessibility improvement is still relatively limited and specific surface area is fairly low^10^,^11^.

As a result of the aforementioned issues (i–iii), supports, such as activated carbon, silica, clay, alumina and zeolite, are used to immobilize photocatalysts^12^,^13^,^14^,^15^,^16^. However, TiO_2_ synthesized on the surface of these supports still sinks without agitation. Then, a new concept of “floatable substrate” was developed. Expanded perlite (EP) as a floating carrier is a light-weight and porous material that can float on the solution surface. It can be combined with SiO_2_ to form the Ti–O–Si bond firm easily. Such photocatalyst improves thermal stability and increases specific surface area^17^. It also enhances the illumination utilization processes in solar irradiation system. Moreover, it enhances the use of photocatalyst oxygenation in the air/water interface, particularly for nonstirred reactions^18^,^19^.

Mesoporous materials with optimal aperture are necessary to address issue (iv). Optimal pore sizes synthesize mesoporous with high specific surface areas and large pore sizes. They also provide a large interface between photocatalysts and organic macromolecules to maintain good transport of organic compounds.

In several studies, the sol–gel approach promotes the formation of porous structures by careful tailoring^20^. Lan *et al*.^21^ reported that the structural and optical properties of films by sol–gel method could be methodically characterized to define the optimal film deposition condition with minimum optical loss.

The preparation of optimized mesoporous materials with large specific surface area and large pore size, and on this basis, controlled co-doping to improve the photocatalytic performance by a simple method is not easy. Meanwhile, materials that possess the characteristics of strong practicability, recoverability, and convenient application are important. Unfortunately, the aforementioned characteristics are still confronted with big challenges. Based on this, a simple method was used to achieve the above characteristics of materials. In this paper, EP was applied to support B–N co-doped TiO_2_ by the sol–gel method with the titanium isopropoxide, boric acid and gaseous ammonia respectively as Ti precursor, B and N source. Optimized mesoporous existed in B–N–TiO_2_/EP with specific surface area of 99.23 m^2^ g^−1^ and pore size of 33.39 nm. The adopted approach was facile and efficient for the synthesis of floating optimized mesoporous photocatalyst with the structure of Ti–O–B–N and O–Ti–B–N. These photocatalysts may provide a potential platform to avoid the redundancy of the hard-template method for photocatalytic oxidation applications (Fig. 1).

## Results

### Characteristics of as-synthesized B–N–TiO_2_/EP

**Figure 2:**
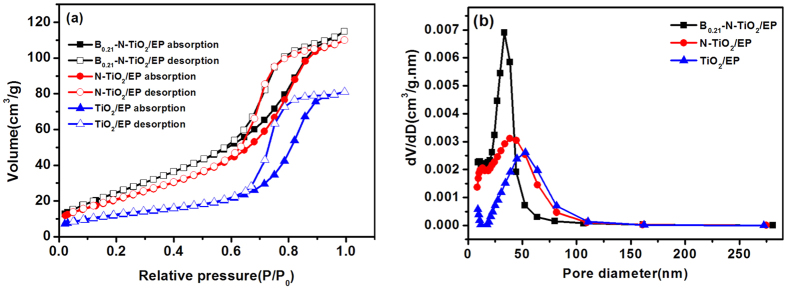
Nitrogen adsorption–desorption isotherm (**a**), and BJH corresponding pore size distribution curve (**b**) of B_0.21_–N–TiO_2_/EP (molar ratio of Ti:B = 1:0.21, 400 ml·min^−1^ ammonia flow), N–TiO_2_/EP, TiO_2_/EP.

Nitrogen adsorption–desorption presents a type–IV isotherm in Fig. 2(a), demonstrating typical mesoporous characteristics of photocatalysts. H1 type hysteresis loops indicate their comparatively large pore sizes^22^. The pore size distribution is calculated using Barrett–Joyner–Halenda (BJH) approach as shown in Fig. 2(b). Ineffective micropores, small amounts of mesoporous, and large amounts of macroporous exist in TiO_2_/EP. The mesoporous materials increased with nitrogen doping and possess pure mesoporous mainly with pore size of 30 nm by B and N co-doping. B_0.21_–N–TiO_2_/EP possesses pure mesoporous, and the composites of mesoporous and macroporous exist in N–TiO_2_/EP and TiO_2_/EP materials. As summarized in Table 1, B_0.21_–N–TiO_2_/EP has a specific surface area of 99.23 m^2^ g^−1^ and a broad pore size distribution centered at 33.39 nm. Therefore, the catalysts are optimized mesoporous materials. Notably, the specific surface area can be significantly increased under ammonia atmosphere, from 44.45 m^2^ g^−1^ to 81.41 m^2^ g^−1^, and the pore volume also increases from 0.1253 cm^3^ g^−1^ to 0.1701 cm^3^ g^−1^; however, the pore diameter decreases from 52.71 nm to 38.58 nm. This effect is possibly attributed to the N doped by gaseous ammonia, which can effectively expand the space and disperse agglomeration. B-doping can also increase the specific surface area of TiO_2_, and the result agrees well with that of Xu *et al*.^23^.

**Figure 3:**
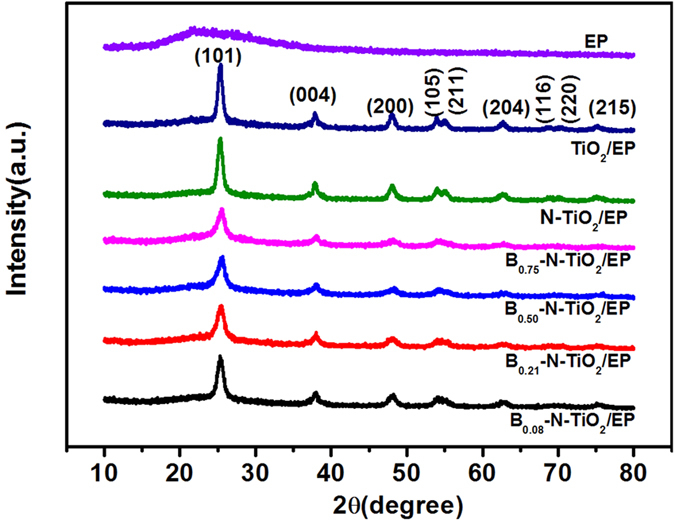
XRD patterns and corresponding diffraction peaks for (**a**) B_0.08_–N–TiO_2_/EP, (**b**) B_0.21_–N–TiO_2_/EP, (**c**) B_0.51_–N–TiO_2_/EP, (**d**) B_0.75_–N–TiO_2_/EP, (**e**) N–TiO_2_/EP, (**f**) TiO_2_/EP. The original EP serves as control.

The XRD patterns of all the synthesized materials are shown in Fig. 3. All the diffraction peaks are demonstrated by pure anatase phase TiO_2_ compared to P25 powder, which is composed of both rutile and anatase phases (80% anatase, 20% rutile), and (001), (003), and (005) are the diffraction peaks of rutile^24^. Among the diffraction peaks of (101) plane, the characteristic peak from B_0.21_–N–TiO_2_/EP shows an evident wide peak. The full width at half maximum value of B_0.21_–N–TiO_2_/EP is approximately 0.704. Given that the ionic radius of B^3+^ is smaller than those of Ti^4+^ and O^2−^, its substitution into the structure of TiO_2_ can increase oxygen vacancies to form surface defects^25^. Notably, B_2_O_3_ is not formed at the surface, which indicates that nitrogen doping reduces the amount of boron into the TiO_2_ crystal lattice. According to the Scherrer equation using the (101) anatase peak, the average crystallite sizes of all samples containing TiO_2_ are approximately 15.5, 15.6, 18.1, 18.0, 18.4, and 20.4 nm, which correspond to (a)–(f); these results are in agreement with the TEM measurement. B and N atoms doping are responsible for the decrease of crystallite sizes caused by the substitution of B or N for O.

SEM images show the morphologies of materials (Figure S1). Nanometer-sized particles are attached to the surface on EP by doping TiO_2_. Figure S1(e) shows that the size of B_0.21_–N–TiO_2_/EP particle is about 30–50 nm. The result confirms that a certain amount of boron doped can induce high surface area associated with the growth inhibition of crystal size^23^.

**Figure 4:**
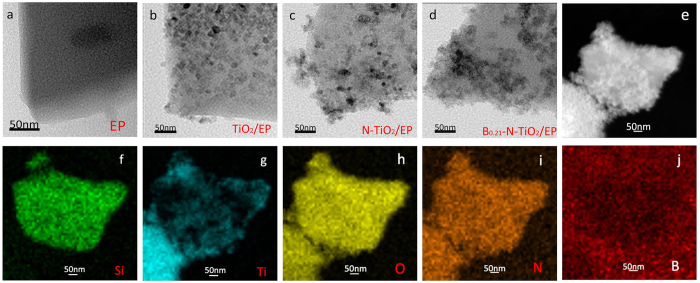
TEM images of (**a**) EP, (**b**) TiO_2_/EP, (**c**) N–TiO_2_/EP and (**d**) B_0.21_–N–TiO_2_/EP; (**e**) HAADF-STEM images of B_0.21_–N–TiO_2_/EP; (**f–j**) the corresponding EDX elemental mapping of silicon, titanium, oxygen, nitrogen, boron.

TEM images show large pore mesoporous materials with high uniformity of rotundity and void sizes (∼30 nm) (Fig. 4b–d), which are in agreement with the results from N_2_ sorption analysis; EP is provided for comparison (Fig. 4a). It can be seen the particles have the uniform size of about 15–20 nm, which are consistent with XRD. High-angle annular dark-field transmission electron microscopy images (Fig. 4e) and elemental mapping images (Fig. 4f–j) of B_0.21_–N–TiO_2_/EP confirmed that Ti, N, and O atoms are uniformly filled and loaded with materials. However, the boron with low atomic number and few content is unrecognized, which corresponds to the result of XPS analysis.

**Figure 5:**
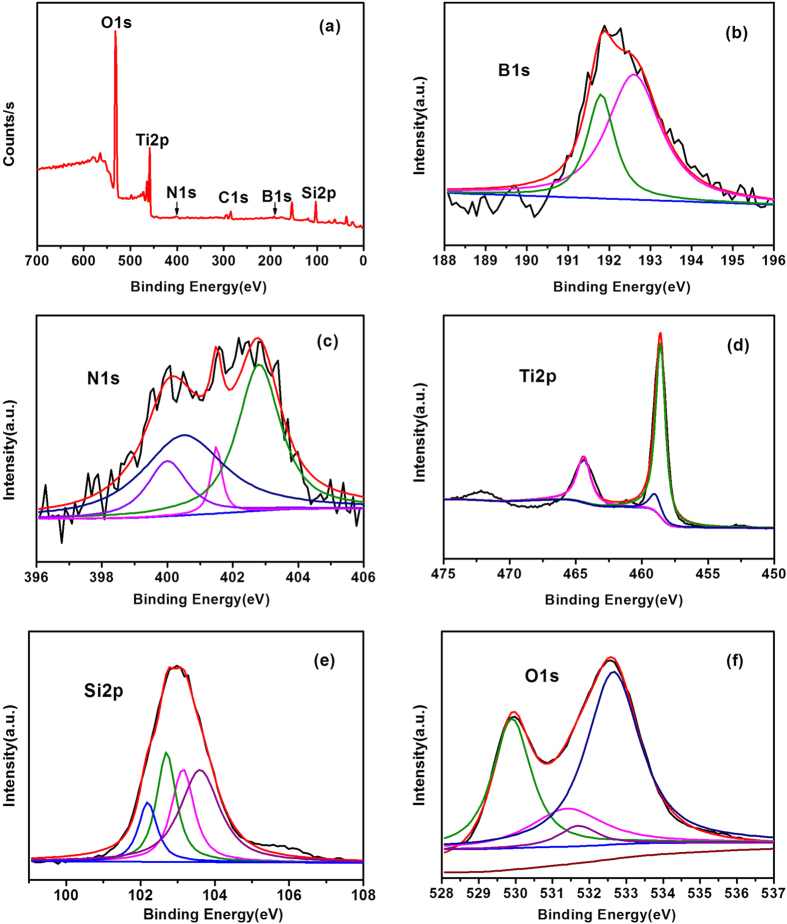
(**a**) XPS spectra of B_0.21_–N–TiO_2_/EP, high-resolution XPS spectra of (**b**) B1s, (**c**) N1s, (**d**) Ti2p, (**e**) Si2p and (**f**) O1s.

The XPS spectra of B_0.21_–N–TiO_2_/EP are shown in Fig. 5. The peaks of oxygen, titanium, nitrogen, carbon, boron and silicon were analyzed in Fig. 5(a). The binding energies of B1s, N1s, Ti2p, Si2p and C1s are 192.18, 401.56, 458.82, 102.97 and 285.2 eV, respectively. The peak of C1s stems from the XPS instrument itself, which contains hydrocarbon and residual carbon of the precursor.

The atomic concentrations of B1s and N1s of the B_0.21_–N–TiO_2_/EP sample from the XPS data are about 4.16 and 2.33 at.%, respectively. The high-resolution XPS spectra for B1s are presented in Fig. 5(b). The peak at 192.6 eV suggests the O–Ti–B and Ti–O–B formed at the TiO_2_ surface, and the content is approximately 69.87%^26^. The B1s peak at 191.8 eV is traceable to the structure of Ti–O–B, and the mass fraction reaches 30.13%^27^. Boron exists in the TiO_2_ crystal lattice mainly in two channels: one is connected with gap, forming a structure of Ti–O–B; and the other is O atom replaced with B atom in the lattice, forming a structure of O–Ti–B^28^,^29^. The high-resolution XPS spectra for N1s are shown in Fig. 5(c) to ensure the structural form of nitrogen. Four peaks at 400.5, 400.0, 401.5 and 402.8 eV are observed. The binding energy *ca.* 400 eV suggests the interstitial incorporation of N elements into the composite material^30^. Hence, the peaks at 400.5 and 400.0 eV are ascribed to the structures of Ti–O–N and Ti–O–B–N (4.75%, 14.11%)^31^, and the peak at 401.5 eV corresponds to the structure of O–Ti–B–N (41.49%)^32^. The peak at 402.8 eV is ascribed to the structure of NH_3_ (39.65%).

Figure 5(d) shows the high-resolution XPS spectra for Ti2p. The peaks of 458.6 eV (2p 3/2) and 464.4 eV (2p 1/2) are attributed to TiO_2_ (TiO_2_–SiO_2_), and the peak at 459 eV is ascribed to TiO_2_ co-doped with B and N atoms^33^. Figure 5(e) presents that the characterization peak at 103.1 eV is the binding energy for normal SiO_2_ or Si–O. The binding energies of O1s are 529.9, 532.65, 528.3, 531.7 and 531.4 eV, which may be attributed to SiO_2_, TiO_2_, Ti–O–B or Ti–O–N, –OH respectively^25^.

### Photocatalytic activities of B–N–TiO_2_/EP samples

**Figure 6:**
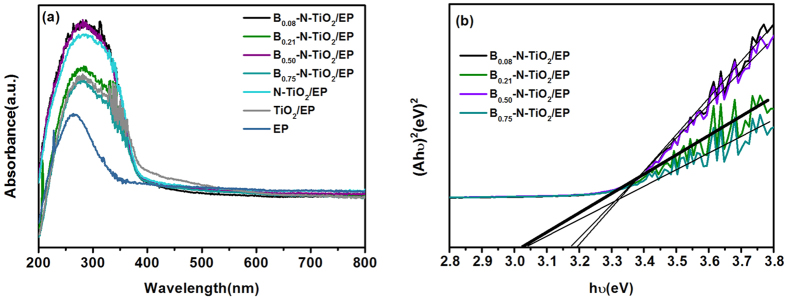
UV–vis absorption spectra (**a**), and band gaps (**b**) of samples.

The UV–vis diffraction reflection spectra (DRS) of (B_m_)-N-TiO_2_/EP samples and control substances are shown in Fig. 6. The synthetic materials exhibit high photo-absorption in UV region (Fig. 6(a)). The TiO_2_/EP has strong absorbance under visible light irradiation (λ > 420 nm). With the doping of nitrogen and boron, the visible light absorptions of the materials obtained become weak and exhibit an evident red shift of absorption. Moreover, the UV–vis DRS of the samples can be used to pinpoint the band gap^34^, as presented in Fig. 6(b), from which a band gap of ∼3.02 eV for B_0.21_–N–TiO_2_/EP is obtained. The value is smaller than B_0.75_–N–TiO_2_/EP (∼3.05 eV), B_0.50_–N–TiO_2_/EP (∼3.18 eV), B_0.08_–N–TiO_2_/EP (∼3.2 eV), and the value of band gap from TiO_2_ anatase should be 3.2 eV. The narrow band gap arises from the B–N co-doped TiO_2_^35^.

**Figure 7:**
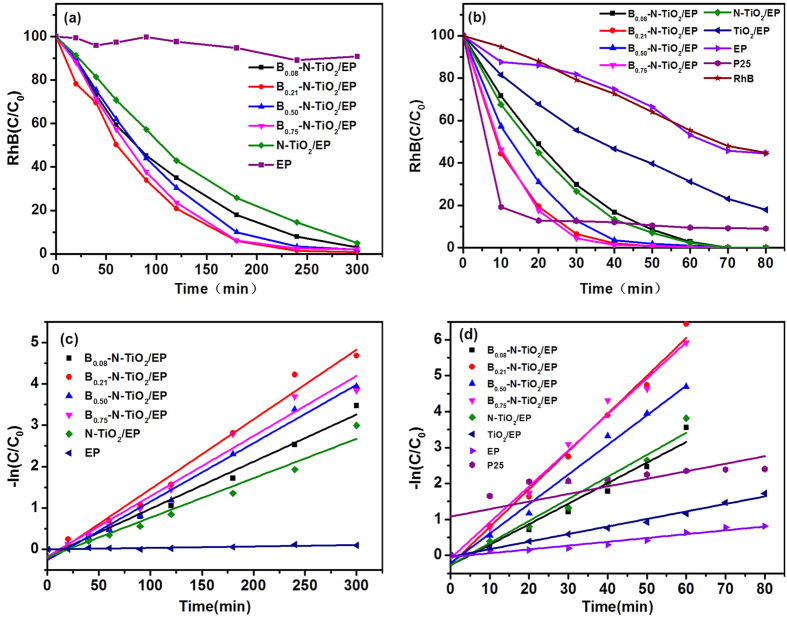
Photocatalytic activities of relevant samples (0.1 g) measured in the 60 ml Rhodamine B (10 mg·L^−1^). (**a**) Visible light irradiation (500 w, Xe lamp); (**b**) UV irradiation (100 w, Hg lamp); (**c**,**d**) Pseudo-first-order kinetics corresponding to (**a**,**b**).

The photodegradation of RhB was evaluated both in UV and visible light region with synthesized materials, and the pseudo-first-order kinetic is shown in Fig. 7. Compared with Fig. 7(a,b), the photodegradation of the samples under UV irradiation is higher than visible irradiation because the absorption is stronger in the UV region (Fig. 7). B/Ti = 0.21 and B/Ti = 0.75 exhibit better performance on photocatalytic activity than the boron content at the level of B/Ti = 0.08 and B/Ti = 0.5, which is attributed to its band gaps; the result is in great agreement with Fig. 6(b). Compared with P25, B–N doped TiO_2_ materials were used obviously to enhance photocatalytic activity after 25 min in the UV region, which is caused by the high specific surface area of the mesoporous structure that enhanced the contact with organic molecules. Furthermore, B–N co-doped with TiO_2_ shows more excellent photocatalytic activity than N–TiO_2_ and TiO_2_.

Figure 7(c,d) show the kinetic studies of the photocatalysts correspond to Fig. 7(a,b). According to the Langmuir–Hinshelwood model, the degradations of RhB follow the pseudo-first-order kinetics. Furthermore, the parameters are recorded through poly fitting the kinetics in Tables S1 and S2, respectively. The k value of B_0.21_–N–TiO_2_/EP is 0.0168 h^−1^ in the visible region, which is 1.77 times larger than N-TiO_2_/EP. In addition, B_0.21_–N–TiO_2_/EP with a k value of 0.1051 h^−1^ is 1.71 times larger than N-TiO_2_/EP, and almost 5 times larger than TiO_2_/EP and P25. The photocatalytic activity of TiO_2_ under visible light is obviously enhanced because of the co-doping of B–N.

**Figure 8:**
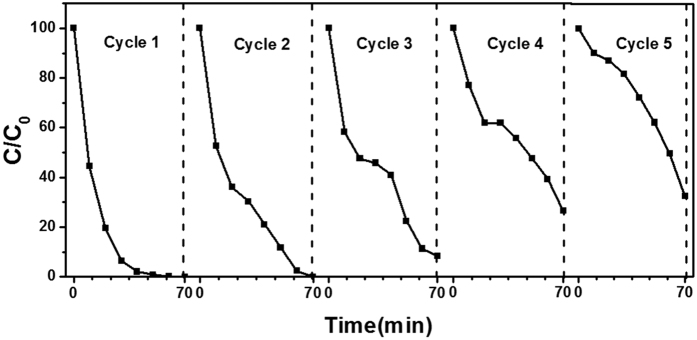
Recycling properties of photocatalytic degradation of RhB over B_0.21_–N–TiO_2_/EP in UV region.

B_0.21_–N–TiO_2_/EP was selected to the five cycles of degradation of RhB under the same condition to probe the recovery of the prepared catalysts. As shown in Fig. 8, the photocatalytic activity of B_0.21_–N–TiO_2_/EP is stable and maintains high photocatalytic performance after three times. The decline occurs in the fourth and fifth reports because of the mass loss of catalyst. Thus, recycling and resourceful utilization of as-synthesis are important.

## Discussions

XRD, BET, SEM, and TEM analyses clearly demonstrate physical properties, namely, crystallinity change, specific surface area, and pore size, and morphological characteristics. Furthermore, XPS and DRS analyses show significant differences, particularly in chemical properties. The co-catalysts during photocatalytic degradation reaction can collect the electrons or holes by strongly promoting the effective transfer of photo-induced carriers and then enhancing the efficiency of the charge-transfer process^29^.

The analysis of photocatalytic degradation for RhB shows the good optical performance of the sol–gel synthesis materials. Compared with the TiO_2_ photocatalyst synthesized by Fan *et al*.^11^ *via* a solvothermal method, which exhibited the best RhB degradation rate of 98.4% (1.916 mg/g) after 120 min (15 w UV lamp), the B_0.21_–N–TiO_2_/EP (m_(TiO_2__)/m_(B_0.21_–N–TiO_2_/EP)_ = 0.24) sample prepared by sol–gel method exhibits better photodegradation rate. The rate reaches 99.8% (6 mg/g) after 60 min (100 w Hg lamp). Furthermore, the RhB photodegradation rate of B_0.21_–N–TiO_2_/EP is 94% under visible irradiation after 3 h, which is better than that of Wang *et al*.^35^ (94%, 1.25 mg/g) under the same condition.

The high photocatalytic activity of the co-catalyst of B–N–TiO_2_/EP can be explained by several reasons. (i) Higher specific surface area and larger pore size are beneficial to the degradation in the reaction and catalytic oxidation on the catalyst surface. However, the materials of large-sized pores with low specific surface area also have low photocatalytic activity, as TiO_2_–EP we obtained. Even the microporous TiO_2_ with high surface area also tend to have an amorphous character, and the micropores easily collapse^36^. Miao *et al*.^37^ compared different specific surface areas and average pore sizes of mesoporous materials. They found that low photodegradation activity for MB existed in materials with large specific surface area and small aperture, as well as in materials with low surface area and large pore. Hence, the mesoporous materials have large specific surface area, relatively large pore size, and regular pore structure, which can degrade larger molecules or groups and perform as good selective catalysts. The optimized mesoporous material we prepared has a pore size of 33.39 nm and an S_BET_ of 99.23 m^2^ g^−1^; the degradation rate of RhB is 93.8% with the UV (100 w, Hg lamp) irradiation after 30 min. By contrast, Zhao *et al*.^38^ proposed that mesoporous anatase TiO_2_ film could have a pore size of 6.7 nm and a BET surface area of 143.37 m^2^ g^−1^, with an RhB (38.32 mg/g) concentration decrease of 92.1% under irradiation with 250 W Hg lamp for 120 min. The optimization material presented in this paper has the advantages of large aperture and specific surface area, which provides better photocatalytic effect and saves time and energy. (ii) The structure of Ti–O–B–N and O–Ti–B–N can improve the separation and transfer of photogenerated carriers and generate local energy level to enhance the absorption of UV and visible light by co-doping B and N^39^. N^−^ generates photoelectricity, while B atom is used as shallow trap to capture electrons^40^. Moreover, the electrons and holes will be dispersed on the catalyst surface. They react with the hydroxyl on the surface and the absorbed water molecules and oxygen molecules to generate active oxygen as •OH and O_2_^−^. (iii) The floating EP provides higher photocatalytic efficiency under special constraining conditions, *i.e.*, no oxygenation and no stirring^41^.

In this study, some limitations exist, such as the lack scale of the variation by doping different contents of boron and the effects of ammonia flow changes on the material properties. The specific photocatalytic mechanism remains to be further studied.

## Conclusions

In summary, floating optimized mesoporous B–N–TiO_2_/EP photocatalyst with high UV and visible light photocatalytic activities is obtained *via* facile large-scale synthesis. Remarkably, synergistic effect exists between co-doping of B–N–TiO_2_ and the structure with appropriate pore and specific surface area. The optimized mesoporous B–N–TiO_2_/EP has high specific surface area and large pore size to maintain good transport and contact between the catalyst and the organic macromolecule. The structure of Ti–O–B–N and O–Ti–B–N formed by B and N co-doped TiO_2_ contribute to the narrowed band gap, the decrease of average crystallite size, and higher photoactivity in UV and visible light region. In addition, the photocatalysts with EP as carrier prevent collisions between nanoparticles, which is attributed to their floating ability, and are convenient for recycling and reclaiming. Our synthesized floating optimized mesoporous photocatalysts co-doped with B and N can enhance the optical response and form more perfect pore structure, which are important for applications in contaminated environments.

## Methods

### Synthesis of the floating photocatalysts

B–N co-doped TiO_2_ coated on EP was synthesized by sol–gel method as follows. TTIP (7 ml) and ethanol (10 ml) were stirred to obtain solution A. H_3_BO_3_ (0.14 g, 0.31 g, 0.73 g, and 1.09 g corresponding to 0.08, 0.21, 0.50, and 0.75 of B/Ti mole ratios) was dissolved into 4 ml of deionized water and 6.5 ml of acetic acid with vibration to obtain solution B. EP (3 g) was dropped into solution A, then a mixture was gained by adding solution B dropwise into solution A. The resultant mixture was slightly oscillated until a white gel was formed. The gel was placed under static condition, aged at room temperature for 24 h, and dried at 80 °C to obtain the xerogel. The xerogel was further nitrided by nitrogen decomposed from gaseous ammonia (NH_3_, 400 ml·min^−1^) at 420 °C in a pipe type oven in the lab. For comparison, N–TiO_2_/EP was prepared without H_3_BO_3_ according to the above procedure. In addition, both H_3_BO_3_ and NH_3_ were absent in the preparation of TiO_2_/EP.

## Additional Information

**How to cite this article**: Xue, H. *et al*. Floating photocatalyst of B-N-TiO_2_/expanded perlite: a sol-gel synthesis with optimized mesoporous and high photocatalytic activity. *Sci. Rep.* **6**, 29902; doi: 10.1038/srep29902 (2016).

## Supplementary Material

Supplementary Information

## Figures and Tables

**Figure 1:**
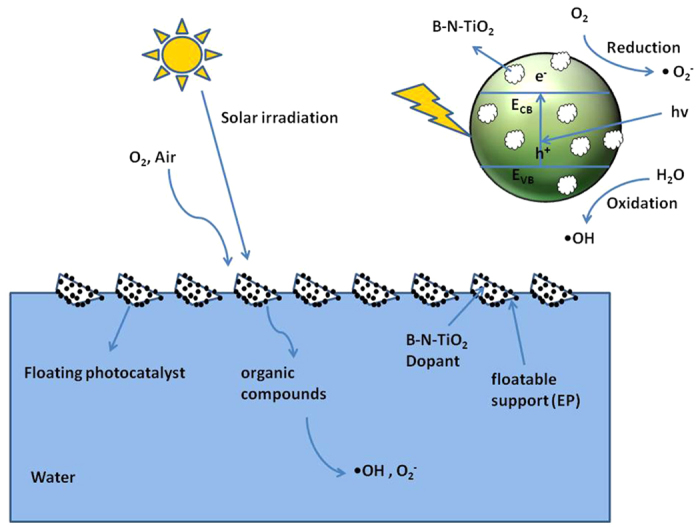
Schematic depiction of the floating optimized mesoporous photocatalyst of B–N–TiO_2_ coated on expanded perlite.

**Table 1:** Characterization results of synthetic samples.

Sample	S_BET_^a^ (m^2^ g^−1^)	Pore size^b^ (nm)	Pore volume^c^ (cm^3^ g^−1^)
B_0.21_-N-TiO_2_/EP	99.23	33.39	0.1778
N-TiO_2_/EP	81.41	38.58	0.1701
TiO_2_/EP	44.45	52.71	0.1253

^a^Surface area was calculated with Brunauer-Emmett-Teller (BET) method.

^b^Estimated from the Barrett-Joyner-Halenda (BJH) formula.

^c^Single point adsorption total pore volume of pores at P/Po = 0.99.
