# Supplementary material for: Floating photocatalyst of B–N–TiO2/expanded perlite: a sol–gel synthesis with optimized mesoporous and high photocatalytic activity
Source: Sci Rep. 2016 Jul 19;6:29902. doi: 10.1038/srep29902 (PMC4949471; doi:10.1038/srep29902)
Supplement: Supplementary Information [file srep29902-s1.pdf]

Supplementary information

Floating photocatalyst of B–N–TiO_2/expanded perlite: a sol–gel synthesis with optimized mesoporous and high photocatalytic activity

Hongbo Xue[1], Ya Jiang[1], Kechun Yuan[1], Tingting Yang[1], Jianhua Hou[1], Chuanbao Cao[2], Ke Feng[1] & Xiaozhi Wang[1]

[1]School of Environmental Science and Engineering, Yangzhou University, Yangzhou 225127, P.R. China. [2]Center of Materials Science, Beijing Institute of Technology, Beijing 100081, P.R. China. Correspondence and requests for materials should be addressed to X. W. (email: xzwang@yzu.edu.cn)

Methods

Materials. Ethanol (C_2H_6O), boric acid (H_3BO_3), acetic acid ($C_2H_4O_2$) and other reagents of analytical grade and titanium (IV) isopropoxide (TTIP, Ti%27.5-28.2, Ourchem) were purchased from Sinopharm Chemical Reagent Beijing Co., Ltd. (China) and used as received without further purification.

The EP was provided by commercial market. Before preparing the composites, the EP was activated by nitric acid and washed to neutral with deionized water, then ultrasonicated for 30 min, dried at 80℃ for 24h. Deionized water was used in all experiments.

Characterization. In order to analyze the crystal structure of photocatalysts more thoroughly, the X-ray diffraction (XRD) pattern from 10°to 80° was measured by Bruker AXS D8 Advance (German) X-ray diffractometer (Cu Kα radiation at 40 kV and 40 mA). Field emission scanning electron microscopy (FESEM) and transmission electron microscopy (TEM) were performed by using Hitachi S-4800 and FEI Tecnai G2 F30 S-TWIN (America), respectively. An Axis Ultra spectrometer was used for XPS analysis. Brunauer-Emmett-Teller (BET) test was performed to measure the specific surface area and pore size distribution by ASAP 2020. The diffuse reflectance spectra (DRS) was measured using UV-VIS-NIR scanning spectrophotometer (Shimadzu UV-2550, Japan) equipped with an integral sphere (200–800 nm) and $BaSO_4$ was the reference sample.

Photocatalytic activity test. The photocatalytic activity of photocatalyst was tested by decomposing RhB dye under both UV and visible light irradiation at a room temperature with XPA-system photochemical reactor from XUJIANG INSTRUMENT, Nanjing. Xe lamp (500w) and Hg lamp (100w) were used as the visible light and UV light, respectively. In the

experimental process, cooling water continuously flows through the lamp shade, there is no heat effect produced during photocatalysis. For the experiments, the concentration of 60ml RhB solution was 10 mg · L^{-1} and 0.1g photocatalyst was added. The mixture was magnetically stirred for 1.5h in the dark before irradiation order to obtain the adsorption–desorption equilibrium solution. After pre-absorption, the samples were taken out in reasonable time and centrifuged at 4000 r · min^{-1} for 10 minutes. The concentration of RhB in remaining clear liquid was analyzed by UV–VIS spectrophotometer (UV-2450, Shimadzu Suzhou Instruments Mfg. Co, Ltd) at 553nm.

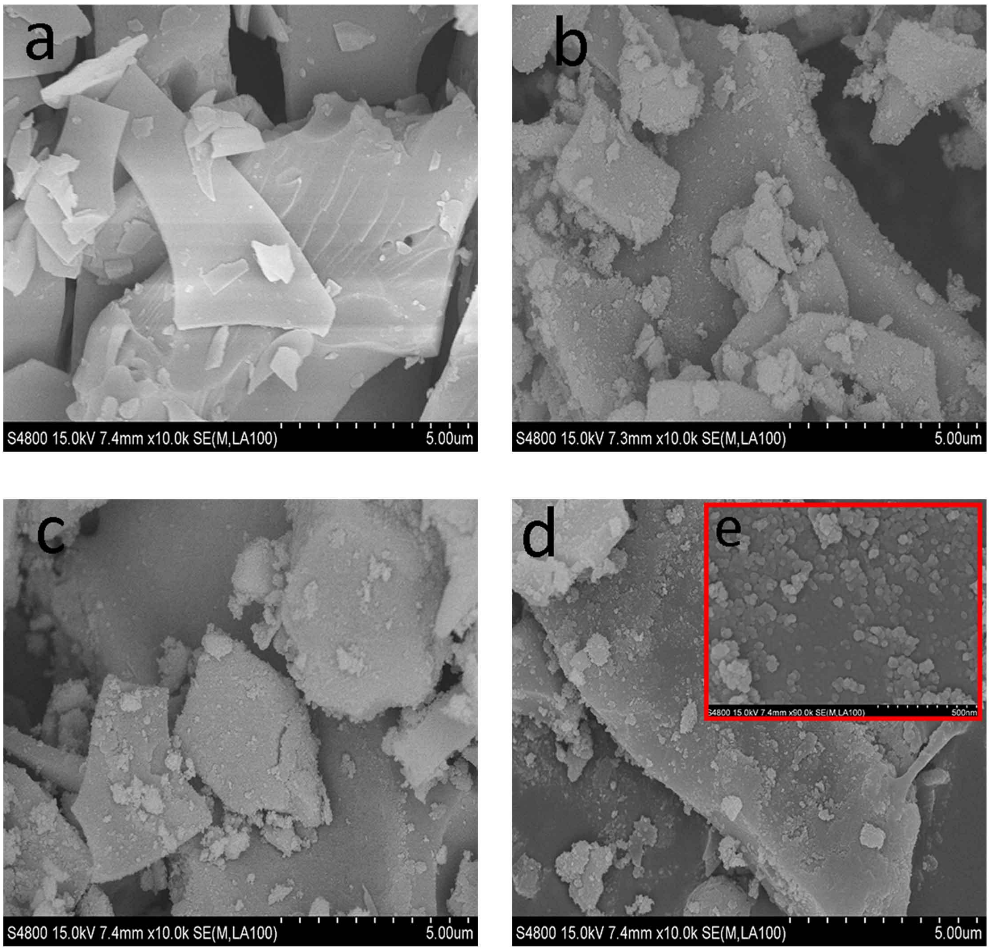

Figure S1. Typical SEM images of (a) EP, (b) TiO_2/EP, (c) N–TiO_2/EP and (d–e) $B_{0.21}$–N–TiO_2/EP.

Table S1 Kinetic parameters of photocatalytic degradation of RhB solution under visible light irradiation by different particles.

Photocatalyst	K/h^{-1}	$t_{1/2}$(h)	R^2
$B_{0.08}$–N–TiO_2/EP	0.0114	0.1634	0.9863
$B_{0.21}$–N–TiO_2/EP	0.0168	0.2259	0.9830
$B_{0.50}$–N–TiO_2/EP	0.0141	0.2576	0.9834
$B_{0.75}$–N–TiO_2/EP	0.0145	0.1676	0.9708
N–TiO_2/EP	0.0095	0.1879	0.9668
EP	0.0003	8.3363E-5	0.7382

Table S2 Kinetic parameters of photocatalytic degradation of RhB solution under UV irradiation by different particles.

Photocatalyst	K/h^{-1}	$t_{1/2}$(h)	R^2
$B_{0.08}$–N–TiO_2/EP	0.0572	0.2765	0.9525
$B_{0.21}$–N–TiO_2/EP	0.1051	0.2574	0.9863
$B_{0.50}$–N–TiO_2/EP	0.0823	0.2165	0.9863
$B_{0.75}$–N–TiO_2/EP	0.1002	0.0825	0.9864
N–TiO_2/EP	0.0613	0.2687	0.9585
TiO_2/EP	0.0210	0.0352	0.9901
EP	0.0106	0.0458	0.9438
P25	0.021	-1.0797	0.5173
